# Preparing for an AI-driven future: insights from Saudi pharmacy students

**DOI:** 10.7717/peerj.20600

**Published:** 2026-01-30

**Authors:** Dalia Almaghaslah, Arwa Khaled, Shadma Wahab, Geetha Kandasamy

**Affiliations:** 1 Department of Clinical Pharmacy, College of Pharmacy, King Khalid University, ABHA, Saudi Arabia; 2 Department of Pharmacogonosy, College of Pharmacy, King Khalid University, ABHA, Saudi Arabia

**Keywords:** AI, Pharmacy students, Saudi Arabia, Artificial intelligence, ChatGPT

## Abstract

**Aim:**

The current study was conducted to assess pharmacy students outlook towards artificial intelligence (AI), pharmacy education, and pharmacy practice.

**Methods:**

The study used a cross-sectional, self-administered, anonymous online questionnaire. The study was conducted at the college of pharmacy in Saudi Arabia.

**Participants:**

Year 4 and year 5 undergraduate pharmacy students were chosen as the study population.

**Results:**

A total of 133 pharmacy students completed the survey (response rate: 82%). The mean Likert score for perceptions of AI use in pharmacy education and practice was 3.43 ± 0.6, while the mean score for incorporation of AI in pharmacy education programs was 3.55 ± 0.78. Students demonstrated generally positive attitudes toward AI, supporting its development and integration into pharmacy curricula, though ethical awareness remained moderate.

**Discussion and Conclusion:**

Most students were supportive of the development of AI in the pharmacy field and valued the importance of having some basic understanding of AI. Ethical and legal consideration of AI used was raised by participants. They were supportive of integrating AI into pharmacy program curricula. Their top-rated learning objectives involved understanding and interpreting AI generated results, gaining awareness of ethical consideration of using AI in clinical practice, comprehend how basic technology process the work, expressing how technology functions in a way that others can grasp.

## Introduction

Digitalization of health has been an important aspect of healthcare that was given attention by the World Health Organization (WHO) (Al-Kahtani et al., 2022). Countries were encouraged to develop and adopt digital health to ensure effective delivery of healthcare services through technology. A Saudi Arabia healthcare transformation program was launched in 2021 under the Vision 2030. The program focused on promoting e-health and digital solutions to improve health accessibility and quality. It also aimed at increasing the numbers of healthcare practitioners, and improving healthcare infrastructure. Achieving the program goals and overcoming the identified challenges require implementing innovative initiatives such as artificial intelligence (AI) (Alasiri & Mohammed, 2022).

AI is defined as “the capacity of machines to carry out tasks that ordinarily require human intelligence, including sensing, thinking, learning, and decision-making” (Saeed, Bin Saeed & AlAhmri, 2023).

The dramatic increase in the utilization of AI in healthcare require healthcare disciplines students to be aware of its principles (Nikitha et al., 2024). Previous literature suggested that health profession students have acceptable understanding of AI concepts, are familiar with the AI concepts and technologies, and regard AI positively (Abdel Aziz et al., 2024; Raza et al., 2022; Roosan, 2023).

AI has been incorporated in various healthcare services including documentation of patients’ profile, diagnosis, surgical assistance, and treatment. AI has also been reported to have explicit roles in the management of specific medical conditions such as radiology, tumors, skin lesions, chest pain, Alzheimer’s disease (Saeed, Bin Saeed & AlAhmri, 2023). Other areas of healthcare that AI has proven to be effective in are medication optimization, decision-making in the management of complex cases with multiple medical conditions and comorbidities (Raza et al., 2022).

Pharmacy disciplines have embraced the use of AI in certain areas including drug design, drug discovery, drug delivery, as well as pharmacy practice (Abdel Aziz et al., 2024).

AI has also been used in pharmacy education such as plagiarism checker, examination integrity, chatbots. The use of AI technology has become a crucial part in the technical and research aspects of pharmacy practice (Syed, Basil & Al-Rawi, 2023). The use of automation in prescription dispensing is an example of how AI addressed the increasing numbers of prescriptions, while facing shortages in the pharmacy workforce. Automated dispensing was found to facilitate workflow, decrease operation costs while maintain safety, accuracy in the pharmacy practice area. AI has also been used in areas including drug-drug interactions, medication therapy management, and drug formulary (Jarab et al., 2023).

A previous study conducted to assess healthcare students’ perceptions towards AI in Jordan highlighted the need to integrate AI topics and to overcome the identified barriers (Al-Qerem et al., 2023). Another study in Qatar concluded that there was a need for more resources to be incorporated to achieve better understanding of AI (Ahmad et al., 2023). A multinational study conducted in the Middle East and North Africa region (MENA) found that Arab healthcare students have limited knowledge and insufficient training, but they exhibited positive views on AI and its implementation in their perspective specialties (Allam et al., 2024).

This study aimed to assess pharmacy students’ perspectives on the role of generative AI tools, such as ChatGPT, which utilize large-scale, general-purpose datasets, in pharmacy education and practice. It explored their readiness to adopt such tools, awareness of ethical implications, and preferences for integrating generative AI-related content into the pharmacy curriculum.

### Artificial intelligence

#### Study design

The study used a cross-sectional design self-administered online questionnaire. The study was conducted between January and April 2023.

#### Settings

The study was conducted at the College of Pharmacy in Saudi Arabia.

#### Participants

Year 4 and year 5 undergraduate pharmacy students were chosen as the study population.

Inclusion criteria were as follows: undergraduate pharmacy program in year 4 or year 5 aged ≥18 years.

### Sample size and sampling procedure

A nonprobability convenience sample was selected. A letter of invitation was sent to all students through the online learning platform (Blackboard), three weeks after the initial invitation, a further reminder was sent through Blackboard. A final reminder was sent after one month. The reason behind selecting this batch of students was that they were more likely to be exposed to different artificial intelligence technologies during the years of their pharmacy programme. Taking part in the study was voluntary, students were encouraged to participate in the study through Blackboard announcements and direct in-person recruitment in the class. The sample size was determined based on the total number of students enrolled in year 4 and year 5 (*n* = 280) and determined using Raosoft sample size calculator (http://www.raosoft.com/samplesize.html) (accessed on 14 February 2023) with a predetermined margin of error of 5% and a confidence level of 95%. In order to minimize erroneous findings and increase study reliability, the target sample size was set at 163.

### Data collection form

The data collection tool was adapted from a previous study with same purpose (Teng et al., 2022).

The questionnaire consisted of four sections. The first section collected student demographics including age, gender, year of study and background information about future career intentions and general outlook on artificial intelligence. The second section asked about the use of artificial intelligence in pharmacy education and pharmacy practice. It contained six items which used a five-point Likert scale ranging from 1 strongly disagree to 5 strongly agree. The third section, asking about incorporation of AI in pharmacy education programs. It contained seven items which used a five-point Likert scale ranging from 1 strongly disagree to 5 strongly agree. Two additional questions collected information about students’ beliefs regarding the time AI will affect pharmacy as a profession and how would they prefer to learn more about AI.

Face and content validity were conducted by a group of experts in the field of pharmacy education. The questionnaire was available in both Arabic and English after conducting back-translation to assure the accuracy of the translation conducted the study author DA who are bilingual speaker of both languages.

The final survey was piloted with four year-5 pharmacy students to ensure the clarity of language and the questionnaire structure. The results of the pilot were not included in the final results. The final data collection tool was administered through a survey document in Blackboard.

### Ethics approval

An ethical clearance was given by the Research Ethics Committee at King Khalid University ECM#2023-204. Written informed consent was obtained from students before participating in the study. Participation was voluntary and students had the right to decline the invitation without any consequences. The gathered data were securely kept by the study authors. The study was conducted in accordance with Declaration of Helsinki.

### Statistical analysis

The collected data were downloaded, coded and entered using the Statistical Package for Social Sciences (SPSS) version 26.0 for Mac. Demographic and background information were described in terms of frequencies and percentages. Students views on the use of AI in pharmacy education and pharmacy practice (six items) used a 5-point Likert scale ranging from 1 (strongly disagree) to 5 (strongly agree). Incorporation of AI in pharmacy education program (seven items) used a 5-point Likert scale ranging from 1 (strongly disagree) to 5 (strongly agree). The distribution of the scale was presented in percentages, as well as the median and interquartile range. The internal consistency and reliability of the scales was assessed using Cronbach’s alpha coefficient, with the minimum recommended level being 0.70.

## Results

The participants’ demographics and basic knowledge about AI, collected through a structured questionnaire, are shown in Table 1. A total of 133 pharmacy students were included in the study. Most participants (59.4%) were in the age group of 21–24, 72.2% of the students were in the fourth level, and 70% were females. When the participants were asked how they preferred to study AI in their curriculum, about 55% wanted it as a continuous workshop during the courses. The participants agreed that AI might affect their careers within 5 or 10 years (36.8 and 36.1%). 43.6% of the participants want to practice clinical work, followed by 33.1% who would like to do research in the future.

**Table 1:** Demographics and background information.

	N	%
Age in years		
Less than 21	38	28.6
21–24	79	59.4
More than 24	16	12
Year of the study		
Fourth level	96	72.2
Fifth level	37	27.8
Gender		
Male	40	30
Female	93	70
When do you think AI will affect your career?		
Within one year	10	7.5
Within 5 Years	49	36.8
Within 10 years	48	36.1
Within 20 years	22	16.5
Will not affect	4	3
How should teaching the basics of artificial intelligence be part of your curriculum or outside the scope of the curriculum?		
Two hours workshop	30	22.6
Continuous workshops	73	54.9
One day course	17	12.8
Ph.D. and master education	13	9.8
Which of the following statements expresses your opinion regarding your future career?		
I would like to do research in my field of specialization in the future	44	33.1
I only want to practice my clinical work	46	43.6
I would like to open my own business/clinic	43	32.3

Table 2 highlights pharmacy students’ perspectives on AI in pharmacy practice, revealing overall positive attitudes with notable areas for improvement. The statement “To what extent do you support the development of artificial intelligence in your field of specialization?” received the highest weighted average (WA) of 3.86, a Median of 4.0, and an interquartile range (IQR) of 3.0–5.0, indicating strong support and enthusiasm for advancing AI in pharmacy. Similarly, the statement “I believe that AI will have an impact on my job” also scored a Median of 4.0, with a narrower IQR of 3.0–4.0 and a WA of 3.33, reflecting optimism about AI’s potential career impact. Students showed moderate agreement on the importance of foundational AI knowledge, as evidenced by the statement “I believe that pharmacy should learn the basics of AI” (Median = 4.0, WA = 3.52, IQR = 3.0–4.0).

**Table 2:** The use of artificial intelligence in pharmacy education and pharmacy practice.

Variable	Strongly agree (%)	Agree (%)	Neutral (%)	Disagree (%)	Strongly disagree (%)	Median	IQR (Range)	Weighted average
To what extent do you support the development of artificial intelligence in your field of specialization?	29.3	39.1	23.3	5.3	3	4.0	3.0–5.0	3.86
I believe that AI will have an impact on my job	14.3	42.1	20.3	9	14.3	4.0	3.0–4.0	3.33
I believe that pharmacy should learn the basics of AI	11.3	48.1	26.3	9.8	4.5	4.0	3.0–4.0	3.51
I am aware of the ethical considerations of the use of AI in my specialty	6.8	21.8	40.6	23.3	7.5	3.0	2.0–4.0	2.96
I feel optimistic about the use of AI in my field	10.5	31.6	33.8	15.8	8.3	3.0	3.0–4.0	3.2
I am concerned about the role AI will play in my field	12.8	36.1	31.6	15	4.5	3.0	3.0–4.0	3.375
I believe that AI is a technology that requires careful management	24.8	39.1	28.6	3.8	3.8	4.0	3.0–4.0	3.77
Mean of weighted average (MWA)						3.55		3.43

However, awareness of ethical considerations surrounding AI was less prominent, with the statement “I am aware of the ethical considerations of the use of AI in my specialty” scoring a median of 3.0, WA of 2.97, and a broader IQR of 2.0–4.0, indicating variability in awareness levels. Optimism about AI use was moderate, as reflected in “I feel optimistic about the use of AI in my field” (median = 3.0, WA = 3.20, IQR = 3.0–4.0), and students expressed balanced concerns about its role in “I am concerned about the role AI will play in my field” (median = 3.0, WA = 3.38, IQR = 3.0–4.0). Strong agreement was observed on the need for oversight, with “I believe that AI is a technology that requires careful management” scoring a median of 4.0, WA of 3.77, and IQR of 3.0–4.0.

The mean weighted average (MWA) across all statements was 3.55, reflecting a general consensus of agreement among students. While students are largely optimistic about AI’s role in pharmacy and support its development, the variability in ethical awareness and concerns about its role highlight areas where targeted education and training could further enhance their preparedness for an AI-integrated future.

Table 3 evaluates students’ perceptions of the importance of incorporating various AI-related objectives into pharmacy education programs. The objective “Understand and interpret results generated by artificial intelligence” received the highest rating, with a weighted average (WA) of 3.68 and an interquartile range (IQR) of 3.0–4.0. This highlights students’ strong agreement and enthusiasm for developing practical skills to analyze AI-generated outputs. Similarly, “It enables you to communicate how technology works in a way that others can understand” and “Knowledge of the ethical implications of using artificial intelligence in clinical contexts” also scored highly, with WAs of 3.59 and 3.62, respectively, and consistent IQRs of 3.0–4.0. These findings suggest that students value effective communication about AI processes and consider ethical awareness a critical component of their education.

**Table 3:** Incorporation of AI in pharmacy education programs.

If your program will introduce the fundamentals of artificial intelligence, evaluate the importance of the following objectives:	Distribution of responses (%)					Weighted average	IQR (Range)	Weighted average
	1	2	3	4	5			
A. Determine when technology is appropriate for a particular clinical context	6.8	15.8	27.1	42.1	8.3	4.0	3.0–4.0	3.29
B. Understand and interpret results generated by artificial intelligence	3.8	4.5	26.3	50.4	15	4.0	3.0–4.0	3.68
C. It enables you to communicate how technology works in a way that others can understand	3.8	3.8	31.6	51.1	9.8	4.0	3.0–4.0	3.59
D. Knowledge of the ethical implications of using artificial intelligence in clinical contexts	4.5	5.3	30.8	42.1	17.3	4.0	3.0–4.0	3.62
E. Understand how basic technological processes work	4.5	3.8	36.8	38.3	16.5	4.0	3.0–4.0	3.58
F. Learn terminology in order to communicate and collaborate with engineers/developers	4.5	9	30.8	42.1	13.5	4.0	3.0–4.0	3.51
Mean of weighted average (MWA)						3.9		3.54

Other objectives, such as “Learn terminology to communicate and collaborate with engineers/developers” (WA = 3.51, IQR = 3.0–4.0) and “Understand how basic technological processes work” (WA = 3.58, IQR = 3.0–4.0), reflect moderate agreement, underscoring the importance of technical literacy for interdisciplinary collaboration. However, the objective “Determine when technology is appropriate for a particular clinical context” scored slightly lower (WA = 3.29, IQR = 3.0–4.0), suggesting that while students acknowledge its importance, they may feel less confident in this area.

Overall, the MWA across all objectives was 3.54, indicating general agreement and positive attitudes toward the proposed learning goals. The findings highlight students’ readiness to engage with AI in pharmacy practice, with a focus on practical applications, ethical considerations, and interdisciplinary communication. These insights can guide the development of AI-related educational content tailored to students’ priorities and perceived gaps in knowledge.

Table 4 shows the distribution of variables being studied. The mean values of the overall scales are 3.43 and 3.548, respectively. Both scales have a Cronbach alpha coefficient of more than 0.7, which indicates inter-item reliability.

**Table 4:** Distribution and internal consistency of overall scales.

Description of scale	Distribution of responses (%)					Mean	SD	Cronbach’s Alpha
	1	2	3	4	5			
The use of artificial intelligence in pharmacy education and pharmacy practice		3	25.6	92.5	100	3.43	0.6	0.7
Incorporation of AI in pharmacy education programs.	3.8	4.5	28.6	82.7	100	3.548	0.78	0.897

## Discussion

The current study was conducted to assess pharmacy students’ views on AI. Student pharmacists were generally hopeful toward AI. The findings indicate a generally positive attitude toward AI among pharmacy students, with strong support for its development and recognition of its relevance in their field. The statement “To what extent do you support the development of artificial intelligence in your field of specialization?” received the highest WA of 3.86, a median of 4.0, and an Interquartile Range (IQR) of 3.0–5.0, highlighting substantial enthusiasm for advancing AI in pharmacy. Similarly, the statement “I believe that AI will have an impact on my job” scored a median of 4.0, with a narrower IQR of 3.0–4.0 and a WA of 3.33, This finding suggests that students generally acknowledged AI’s potential influence on their professional roles, indicating cautious optimism rather than strong enthusiasm about its impact. Additionally, students expressed moderate agreement on the importance of foundational AI knowledge, underscoring an acknowledgment of its growing significance in pharmacy education and practice. These results suggest that while students are enthusiastic about AI’s integration and its career implications, there may still be room to strengthen their understanding of foundational AI concepts to fully prepare them for its practical applications.

However, they expressed caution regarding the management of AI in the field and reported limited awareness of the ethical consideration involved.

A similar mixed feeling was expressed by Canadian healthcare students, including pharmacists, where they were optimistic towards utilizing AI in their clinical practice, but they were worried that AI will result replacing healthcare professionals (Teng et al., 2022). A recent commentary suggested a reassuring statement; “AI will not replace you, a person using AI will” and encouraged academic institutions to offer informational and training workshops to their students and staff (Cain, Malcom & Aungst, 2023). Equipping pharmacy students and academics with the knowledge and skills required to use AI effectively, will prepare them to be better positioned to survive in an AI-driven world rather reducing the risk lose their roles as pharmacists and consequently losing their jobs.

Pharmacy students from 12 different countries including Saudi Arabia, conveyed positive views towards incorporation of AI in daily pharmacy practice. However, they expressed lacking confidence in their AI knowledge and showed enthusiasm for integrating AI education as part of their pharmacy degree program. Ethical and legal concerns were also highlighted by pharmacy students (Allam et al., 2024). Other studies raised the same concerns regarding ethical and legal aspects of using AI in pharmacy curricula and pharmacy practice. Studies suggested establishing ethical guidelines and legal framework that control AI-driven recommendations (Hasan et al., 2024b; Mortlock & Lucas, 2024; Chalasani et al., 2023; Hasan et al., 2024a).

In the current study, pharmacy students were supportive of integrating AI into pharmacy program curricula. Their top-rated learning objectives involved understanding and interpreting AI generated results, gaining awareness of ethical consideration of using AI in clinical practice, comprehend how basic technology process the work, expressing how technology functions in a way that others can grasp. Other objectives involved; Being able to assess the appropriateness of utilizing AI in certain clinical contexts, acquire the terminology to effectively communicate with engineers and developers. A previous study reported sharing the same top-ranked objectives of AI incorporation into pharmacy degree program. For that reason, designing an AI course should consider meeting the highest-rated objectives (Allam et al., 2024).

Similar findings were expressed in a recent multi-country study that highlighted the importance of incorporation of AI-based courses into pharmacy program and continuing medical education. They emphasized the significance of early exposure to AI to advance knowledge and skills required in real-world clinical scenarios (Allam et al., 2024) .

To address the identified gaps, pharmacy curricula should integrate focused modules on AI ethics, covering critical areas such as data privacy, algorithmic bias, accountability in AI decision-making, and the potential consequences of AI errors in clinical contexts. Case-based learning can be utilized to provide students with real-world scenarios for applying ethical principles in decision-making. Interdisciplinary collaboration with AI developers and ethicists can further deepen students’ understanding of how ethical considerations are embedded into AI systems. Coupled with practical exposure to AI tools and discussions on their ethical implications, these strategies will equip students to critically assess AI-driven recommendations and advocate for their responsible application in pharmacy practice. Embedding ethical training within the curriculum will help students develop the competencies required to manage AI responsibly, bridging the gap between technical expertise and ethical awareness. These efforts will not only enhance students’ confidence in using AI but also ensure they are prepared to tackle the ethical challenges associated with its integration into pharmacy.

To ensure practical readiness, the curriculum must extend beyond theoretical knowledge to include hands-on experience with AI tools such as clinical decision-support systems and automated dispensing technologies. Modules should address AI applications in drug development, clinical decision-making, and ethical considerations, fostering interdisciplinary collaboration skills. Practical training is vital to complement theoretical understanding, enabling students to critically evaluate AI outputs and apply them effectively in real-world pharmacy settings.

Recent research assessed pharmacy students’ familiarity with chat-based AI tools, such as ChatGPT^®^, and their usage patterns. The study found that 88% of students were familiar with these tools, primarily utilizing them for assignments and studying. While 85.3% believed AI enhances academic performance, concerns about potential distractions (65.7%) and academic dishonesty (65.1%) were also noted. The authors emphasized the need to integrate AI education into the pharmacy curriculum to address knowledge gaps and better prepare students for technological advancements (Orok et al., 2024).

Similarly, Culp et al. (2024) reported that only 25% of pharmacy students in a US School of Pharmacy agreed that they were knowledgeable about AI tools, with 50% remaining neutral and 25% disagreeing. In contrast, participants in the present study demonstrated slightly greater awareness and confidence regarding AI use in pharmacy, as indicated by higher agreement levels across most items. This difference may reflect the increasing integration of digital health and AI topics within Saudi pharmacy education under Vision 2030, as well as growing exposure to generative AI tools such as ChatGPT since 2023 (Culp et al., 2024).

Another pertinent study explored pharmacy students’ understanding and attitudes toward AI and machine learning (ML). The study highlighted that while students recognize the potential of AI in enhancing pharmacy practice, there is a significant need for educational interventions to improve their knowledge and confidence in utilizing these technologies effectively (Zhang et al., 2024).

These studies collectively underscore the growing interest and perceived importance of AI among pharmacy students. They also highlight the necessity for curriculum development to incorporate AI education, ensuring that future pharmacists are well-equipped to leverage these technologies in their professional practice (Zhang et al., 2024).

The findings of this study are subject to several limitations. First, the survey was conducted between January and April 2023. Given the rapid evolution of artificial intelligence and the changing perceptions surrounding its use in education, the findings may not fully reflect current attitudes or developments. Second, there was an overrepresentation of female participants and an underrepresentation of males, which may have influenced the generalizability of the results to a more balanced gender demographic. Third, the cross-sectional design limits the ability to establish causal relationships or track changes in perceptions over time. Fourth, the study was conducted at a single pharmacy college in Saudi Arabia, which restricts the generalizability of the findings to similar educational and cultural contexts. Fifth, the relatively small sample size of 133 participants, although adequate for descriptive analysis, may limit the applicability of the findings to the broader population of pharmacy students in Saudi Arabia. The use of a nonprobability convenience sampling method may have introduced selection bias and affected sample diversity, as students more interested in AI or motivated to participate might have been overrepresented. Additionally, reliance on self-reported data introduces the possibility of social desirability bias, where participants may have provided favorable rather than entirely accurate responses.

The survey’s online format, while efficient, may have influenced participation and introduced variability in responses, particularly among students with limited technological access or language proficiency. Although the questionnaire was available in both Arabic and English, students with limited proficiency in either language may have been unintentionally excluded, potentially affecting inclusivity. Moreover, the study did not account for prior exposure to AI technologies or knowledge levels, which could have influenced perceptions. These limitations highlight the need for future studies with larger, more diverse, and multi-institutional samples, incorporating longitudinal and mixed-methods approaches to capture evolving attitudes toward AI integration in pharmacy education and practice.

## Conclusion

The current study was conducted to provide a snapshot of pharmacy students towards the use of AI in pharmacy education and pharmacy practice. Majority of students were supportive of the development of AI in the pharmacy field and valued the importance of having some basic understanding of AI. Ethical and legal consideration of AI used was raised by participants. They were supportive of integrating AI into pharmacy program curricula. Their top-rated learning objectives involved understanding and interpreting AI generated results, gaining awareness of ethical consideration of using AI in clinical practice, comprehend how basic technology process the work, expressing how technology functions in a way that others can grasp.

##  Supplemental Information

10.7717/peerj.20600/supp-1 Supplemental Information 1 Comparison with of Teng et al. (2022) among Canadian pharmacy students

10.7717/peerj.20600/supp-2 Supplemental Information 2 Raw data

10.7717/peerj.20600/supp-3 Supplemental Information 3 Questionnaire

10.7717/peerj.20600/supp-4 Supplemental Information 4 STROBE

## Additional Information and Declarations

### Data Availability

The following information was supplied regarding data availability: The raw data is available in the Supplementary File.
