# Supplementary material for: Preparing for an AI-driven future: insights from Saudi pharmacy students
Source: PeerJ. 2026 Jan 30;14:e20600. doi: 10.7717/peerj.20600 (PMC12863167; doi:10.7717/peerj.20600)
Supplement: Supplemental Information 1 [file peerj-14-20600-s001.docx]

Comparison with of Teng et al. (2022) among Canadian pharmacy students

| **Item** | **Weighted Average (Saudi Study)** | **Median (IQR)** | **Weighted Average – Canadian Pharmacy Students *(Teng et al., 2022)*** | **Comparison Comment** |
| --- | --- | --- | --- | --- |
| **1. Support the development of AI in the field** | **3.86** | 4.0 (3–5) | 4.2 | Similar high support for AI development in both cohorts |
| **2. AI will have an impact on my job** | **3.33** | 4.0 (3–4) | 3.9 | Slightly higher perceived impact among Canadian students |
| **3. Pharmacists should learn the basics of AI** | **3.51** | 4.0 (3–4) | 4.0 | Consistent agreement on the need for AI literacy |
| **4. Aware of the ethical considerations of AI use** | **2.96** | 3.0 (2–4) | 3.5 | Greater ethical awareness among Canadian students |
| **5. Optimistic about the use of AI in pharmacy** | **3.20** | 3.0 (3–4) | 3.8 | More optimism observed among Canadian students |
| **6. Concerned about AI’s role in the field** | **3.38** | 3.0 (3–4) | 3.2 | Comparable moderate concern levels |
| **7. AI requires careful management** | **3.77** | 4.0 (3–4) | 4.1 | Both groups acknowledge the need for responsible oversight |

**Mean Weighted Average (Saudi Study): 3.55**  **Mean Weighted Average (Teng et al., 2022): 3.81**

As shown in Table 2, pharmacy students exhibited generally positive attitudes toward AI, particularly in supporting its development and recognizing the need for responsible management. These results parallel the findings of Teng et al. (2022) among Canadian pharmacy students, though ethical awareness and optimism were slightly higher in the Canadian cohort.
