# Supplementary material for: Preparing for an AI-driven future: insights from Saudi pharmacy students
Source: PeerJ. 2026 Jan 30;14:e20600. doi: 10.7717/peerj.20600 (PMC12863167; doi:10.7717/peerj.20600)
Supplement: Supplemental Information 3 [file peerj-14-20600-s003.docx]

**Questionnaire: Pharmacy Students’ Perceptions of Artificial Intelligence (AI) in Education and Practice**

### **Section 1: Demographics and Background Information**

1. Age in years:
   - Less than 21
   - 21–24
   - More than 24
2. Year of the study:
   - Fourth level
   - Fifth level
3. Gender:
   - Male
   - Female
4. When do you think AI will affect your career?
   - Within 1 year
   - Within 5 years
   - Within 10 years
   - Within 20 years
   - Will not affect
5. How should teaching the basics of AI be integrated into your curriculum?
   - Two hours workshop
   - Continuous workshops
   - One-day course
   - Part of postgraduate (MSc/PhD) education
6. Which of the following statements expresses your opinion regarding your future career?
   - I would like to do research in my field of specialization in the future
   - I only want to practice my clinical work
   - I would like to open my own business/clinic

### **Section 2: The Use of AI in Pharmacy Education and Practice**

(Please indicate your level of agreement with each statement on a 5-point Likert scale: 1 = Strongly disagree, 2 = Disagree, 3 = Neutral, 4 = Agree, 5 = Strongly agree)

1. I support the development of artificial intelligence in my field of specialization.
2. I believe that AI will have an impact on my job.
3. I believe that pharmacy students should learn the basics of AI.
4. I am aware of the ethical considerations of using AI in my specialty.
5. I feel optimistic about the use of AI in my field.
6. I am concerned about the role AI will play in my field.
7. I believe that AI is a technology that requires careful management.

### **Section 3: Incorporation of AI in Pharmacy Education Programs**

(Please rate the importance of each objective if your program introduces the fundamentals of AI: 1 = Not important at all, 5 = Very important)

1. Determine when technology is appropriate for a particular clinical context.
2. Understand and interpret results generated by artificial intelligence.
3. Communicate how technology works in a way that others can understand.
4. Understand the ethical implications of using artificial intelligence in clinical contexts.
5. Understand how basic technological processes work.
6. Learn terminology to communicate and collaborate with engineers/developers.
